# Patients’ impressions of after-hours house-call services during the COVID-19 pandemic in Japan: a questionnaire-based observational study

**DOI:** 10.1186/s12875-021-01534-5

**Published:** 2021-09-15

**Authors:** Kojiro Morita, Ryota Inokuchi, Xueying Jin, Masatoshi Ishikawa, Nanako Tamiya

**Affiliations:** Department of Health Services Research, University of Tsukuba, 1-1-1 Tenno-dai, Tsukuba, Ibaraki 305-8575 Japan

**Keywords:** After-hours house call, Emergency department, Out-of-hour service, Telephone triage

## Abstract

**Background:**

Access to healthcare has been strongly affected by the coronavirus disease 2019 (COVID-19) pandemic, which has raised concerns about the increased risk of delays in receiving medical care. This study aimed to assess the patients’ impressions of after-hour house-call (AHHC) medical services during the COVID-19 pandemic using a patient questionnaire.

**Methods:**

This was a cross-sectional observational study of anonymized medical record data and internet-based questionnaires from patients who used AHHC medical services from April 2020 to January 2021. We summarized the patients’ impressions of AHHC medical services during the COVID-19 pandemic stratified by patient characteristics. The questions of the questionnaire were as follows: (i) Did you use the AHHC medical services because you suspected you had COVID-19 infection? (ii) Do you feel that the use of AHHC medical services has helped prevent transmission of COVID-19? (iii) What action would you have taken in the absence of AHHC medical services?

**Results:**

A total of 1802 patients responded to the questionnaire (response rate: 11.3%).

First, 700 (40.8%) of the responders indicated that they had used AHHC medical services because of suspicion of COVID-19. Second, most responders (88.8%) felt that AHHC medical services prevented transmission of COVID-19. Third, 774 (43.0%) of the responders considered that they would have visited an emergency department or called an ambulance if AHHC medical services had not been used. Furthermore, 411 (22.8%) of the responders indicated that they would remain at home or wait until working hours if AHHC medical services were not available despite having a condition that required emergency attention.

**Conclusions:**

AHHC medical services may be one of the strategies for those who refrain from seeking healthcare services, thus reducing the risk of delayed hospital visits during emergencies. Furthermore, AHHC medical services may also contribute to preventing transmission of COVID-19 by avoiding contact with other patients in the hospital.

## Background

Access to healthcare has been strongly affected by the coronavirus disease 2019 (COVID-19) pandemic. Several studies have revealed a significant reduction in emergency department (ED) visits and have raised concerns about the increased risk of delays in receiving hospital care for emergencies [1–5]. One of the reasons why patients have been refraining from visiting healthcare services has arisen from fear of COVID-19 infection while visiting hospitals or clinics [1, 6, 7].

There are several models for out-of-hour care [8–11], and after-hour house-call (AHHC) medical services are services for semi-urgent cases or cases in which access to healthcare is difficult. In Tokyo, Japan, private AHHC medical services, which send doctors directly to patients’ homes instead of an ambulance, have been available since 2016. This allows patients to receive medical services in their homes even if they do not have a family doctor. The AHHC service is not linked to any specific secondary care/Emergency Department (ED) services. The AHHC service will also directly request medical treatment from any hospital or call an ambulance if urgent treatment is needed. Before the establishment of the AHHC medical service, when a complaint occurs out-of-hours and patients were unable to arrange for a family doctor, patients are generally free to choose medical services as follows: (i) call an ambulance; (ii) visit to the ED by themselves; (iii) call a home clinic or hospital; (iv) call the telephone consultation service; or (v) wait until working hours.

Under the universal healthcare system, Japanese citizens have free access to public or even private medical facilities at any time, which allows people to visit any medical institution(s), such as hospitals or primary healthcare/family medicine facilities, regardless of their symptoms [12]. Furthermore, anyone can call an ambulance for free [13]. However, during out-of-hours periods (nights or holidays), most people cannot access their preferred medical facilities because these facilities do not always operate [14]. The emergency medical system is categorized into primary, secondary, and tertiary hospitals [15]. Generally, primary hospitals provide medical care for patients with mild symptoms who do not need hospital admission and can return home. Secondary hospitals provide emergency care for patients who potentially require hospital admission. Tertiary hospitals offer intensive care for critically ill patients who cannot be treated at primary or secondary emergency hospitals, such as acute myocardial infarction, stroke, or multiple injuries [15, 16]. When patients use an ambulance, they are transported to secondary or tertiary hospitals depending on the severity of their condition. On the other hand, outpatients are also free to access primary, secondary, and even tertiary hospitals during out-of-hours periods.

It is important to understand the potential impact of effective AHHC medical services during the COVID-19 pandemic. AHHC medical services may also reduce the fear of acquiring COVID-19 infection in a hospital or clinic; however, there have been no questionnaire surveys to ask patients about their impressions of AHHC medical services during the COVID-19 pandemic. Therefore, we assessed the patients’ impressions of AHHC medical services during the COVID-19 pandemic using questionnaires after AHHC medical services were used.

## Methods

This was a cross-sectional observational study of anonymized medical record data and internet-based questionnaires from patients who used AHHC medical services from April 2020 to January 2021. The research ethics committee of the University of Tsukuba approved this study [approval number: 1527].

### Healthcare system in Japan

#### AHHC medical services in Japan

In Japan, public AHHC medical services are not available. Fast Doctor Ltd. (Shinjuku, Tokyo, Japan) has been operating as a private AHHC medical service in Tokyo since 2016, sending doctors to a patient’s home during out-of-hours periods (18:00–6:00 on weekdays, 18:00–6:00 on Saturdays, and 24 h a day on Sundays and public holidays).

Patients can call AHHC medical services by phone. AHHC medical services have adopted the telephone triage system developed in the Tokyo Metropolitan Emergency Telephone Consultation Center of the Tokyo Fire Department [17]. Triage level is classified into five categories as follows: (i) red, a condition requiring transport to secondary or tertiary emergency hospitals with an ambulance; (ii) orange, a condition requiring immediate medical consultation at a secondary emergency hospital; (iii) yellow, a condition requiring medical consultation that might worsen; (iv) green, a non-urgent condition requiring primary care; and (v) white, a condition that can be observed at home [18]. AHHC medical services provide home-visit medical services only to patients classified as orange, yellow, and sometimes red based on the triage of the telephone consultation center.

Under the universal health insurance system in Japan, the health care/medical procedures are set in a national fee schedule, and patients pay the same price for the same medical care, regardless of which medical services they access. Patients who use AHHC medical services will be billed for transportation costs (up to about US$9) in addition to their co-pay for the medical consultation.

### Data sources

The study used anonymized medical record data and data of patient questionnaires conducted after patients had used AHHC medical services.

The medical records included data on age, sex, telephone triage levels, primary diagnosis (using the International Classification of Diseases, Tenth Revision), disease severity, prescription of any drugs, time of medical examination, examination by health care personnel wearing personal protective equipment (PPE), screening with a diagnostic tool for COVID-19, and date of services. Reverse transcription-polymerase chain reaction [RT-PCR] for severe acute respiratory syndrome coronavirus 2 [SARS-CoV-2] or rapid antigen test for detection of SARS-CoV-2 had been used as a diagnostic tool for COVID-19 by doctors during medical examinations.

The disease severity was categorized into three levels based on the doctor’s assessment after medical examinations as follows: (i) mild, a condition that could be treated with over-the-counter medications; (ii) moderate, a condition that required an ED visit if not using AHHC medical services; or (iii) severe, a condition that required an ambulance call if not using AHHC medical services.

AHHC medical service providers have been conducting an internet-based patient questionnaire survey about patients’ impressions of AHHC medical services during the COVID-19 pandemic. The questionnaire consisted of three questions as follows: (i) Question on suspicion of COVID-19: “Did you use the AHHC medical services because you suspected you had COVID-19 infection? (Yes or No)”; (ii) Question on perception on prevention of transmission of COVID-19:” Do you feel that the use of AHHC medical services has helped prevent transmission of COVID-19? (Yes or No)”; and (iii) Question on possible actions if AHHC medical services were not available:” What action would you have taken in the absence of AHHC medical services (stay home, wait for consultation until a hospital opens, visit the ED, or call an ambulance)?”.

The patients themselves or their caregivers responded to the questionnaire.

### Study population

We selected AHHC medical service users whose medical record data and data of patient questionnaires existed from April 11, 2020, to January 31, 2021. In Japan, to combat the COVID-19 outbreak, the Japanese government proclaimed an emergency declaration from April 8, 2020, for Tokyo and six other prefectures. The state of the emergency declaration was expanded to the entire country on April 16, 2020. Under Japanese law, the government does not have the authority to implement a lockdown. However, based on this declaration, the government requested to refrain from going out of the house unnecessarily, closed various offices, businesses or schools, and encouraged home working [19].

### Statistical analysis

First, we compared the patient characteristics (age, sex, first triage color, diagnosis, disease severity, prescription of any drugs, examination by health care personnel wearing PPE, diagnostic tools for COVID-19, and time of medical examination) between questionnaire responders and non-responders. Pearson’s chi-square test was used to compare the proportions of categorical variables between the groups. The t-test was used to compare the time of medical examination, a continuous variable.

Second, we summarized patients’ impressions of AHHC medical services during the COVID-19 pandemic stratified by patient characteristics.

All statistical analyses were conducted using Stata/MP version 14 (Stata Corp., College Station, TX, USA), and the level of significance was set at p < 0.05.

## Results

A total of 15,998 patients used AHHC medical services during the study period. A total of 1802 patients responded to the patient questionnaire (response rate: 11.3%).

### Comparison of patient characteristics between questionnaire responders and non-responders

A total of 10,595 patients (66.2%) were aged ≥ 16 years, and the triage color of 8548 patients’ was orange (53.4%). The top three primary diagnostic categories were respiratory, infectious, and digestive diseases. A total of 8775 patients who used AHHC medical services (54.9%) had a condition that could be treated with over-the-counter drugs, and 310 patients (1.9%) would have required an ambulance had AHHC medical services not been used. Drug prescriptions from AHHC medical services were provided to 81.6% of the patients, and the average medical examination time was 29.4 min. A total of 50.3% of patients were examined by health care personnel wearing PPE, and 21.9% of patients were screened using diagnostic tools for COVID-19 (Table 1). Overall, 759 (4.7%) patients were diagnosed with COVID-19 (data not shown).

**Table 1:** Patient characteristics in responders and non-responders

	All patients		Responders		Non-responders		
	(n = 15,998)		(n = 1802)		(n = 14,196)		p
Age (years), n (%)							< 0.001
0–15	5403	(33.8)	618	(34.3)	4785	(33.7)	
16–64	9235	(57.7)	970	(53.8)	8265	(58.2)	
65–74	498	(3.1)	86	(4.8)	412	(2.9)	
> 74	862	(5.4)	128	(7.1)	734	(5.2)	
Male sex, n (%)	7662	(47.9)	832	(46.2)	6830	(48.1)	0.12
Telephone triage color, n (%)							0.2
Yellow	6230	(38.9)	722	(40.1)	5508	(38.8)	
Orange	8548	(53.4)	930	(51.6)	7618	(53.7)	
Red	1220	(7.6)	150	(8.3)	1070	(7.5)	
Diagnosis category, n (%)							0.054
Infectious and parasitic diseases	3201	(20.0)	346	(19.2)	2855	(20.1)	
Endocrine, nutritional, and metabolic diseases	84	(0.5)	15	(0.8)	69	(0.5)	
Mental and behavioral disorders	78	(0.5)	8	(0.4)	70	(0.5)	
Diseases of the nervous system	182	(1.1)	31	(1.7)	151	(1.1)	
Diseases of the ear and mastoid process	182	(1.1)	31	(1.7)	151	(1.1)	
Diseases of the circulatory system	151	(0.9)	17	(0.9)	134	(0.9)	
Diseases of the respiratory system	8897	(55.6)	985	(54.7)	7912	(55.7)	
Diseases of the digestive system	700	(4.4)	77	(4.3)	623	(4.4)	
Diseases of the skin and subcutaneous tissue	421	(2.6)	39	(2.2)	382	(2.7)	
Diseases of the musculoskeletal system and connective tissue	467	(2.9)	51	(2.8)	416	(2.9)	
Diseases of the genitourinary system	537	(3.4)	64	(3.6)	473	(3.3)	
Symptoms, signs, and abnormal clinical and laboratory findings, not elsewhere classified	501	(3.1)	62	(3.4)	439	(3.1)	
Injury, poisoning, and other certain consequences of external causes	559	(3.5)	69	(3.8)	490	(3.5)	
Other	38	(0.2)	7	(0.4)	31	(0.2)	
Symptom severity, n (%)							0.78
Mild	8775	(54.9)	980	(54.4)	7795	(54.9)	
Moderate	6913	(43.2)	784	(43.5)	6129	(43.2)	
Severe	310	(1.9)	38	(2.1)	272	(1.9)	
Prescription of any drugs, n (%)	13,049	(81.6)	1,439	(79.9)	11,610	(81.8)	0.049
Personal protective equipment used, n (%)	8047	(50.3)	965	(53.6)	7082	(49.9)	0.003
Diagnostic tools for COVID-19 used	3508	(21.9)	451	(25.0)	3057	(21.5)	< 0.001
Time of medical examination (minutes), mean (SD)	29.4	(13.6)	29.8	(14.2)	29.4	(13.6)	0.24
Time of medical examination (minutes), n (%)							
< 15	2530	(15.8)	276	(15.3)	2254	(15.9)	0.78
16–25	5576	(34.9)	625	(34.7)	4951	(34.9)	
≥ 26	7892	(49.3)	901	(50.0)	6991	(49.2)	

*Abbreviations*: *AHHC* after hour house call, *COVID-19* coronavirus disease 2019, *ED* emergency department, *SD* standard deviation

Responders to the questionnaire were more likely to be older (mean age (years [± standard deviation]): 31.3 [± 24.9] vs. 27.4 [± 22.6]; p < 0.001), less likely to receive any drug prescription (79.9% vs. 81.8%; p = 0.049), more likely to be examined by health care personnel wearing PPE (53.6% vs. 49.9%; p = 0.003), and more likely to be screened with diagnostic tools for COVID-19 (25.0% vs. 21.5%; p < 0.001) than non-responders. However, there were no clinically significant differences between the two groups (Table 1).

### Patients’ impressions of AHHC medical services during the COVID-19 pandemic

**Fig. 1:**
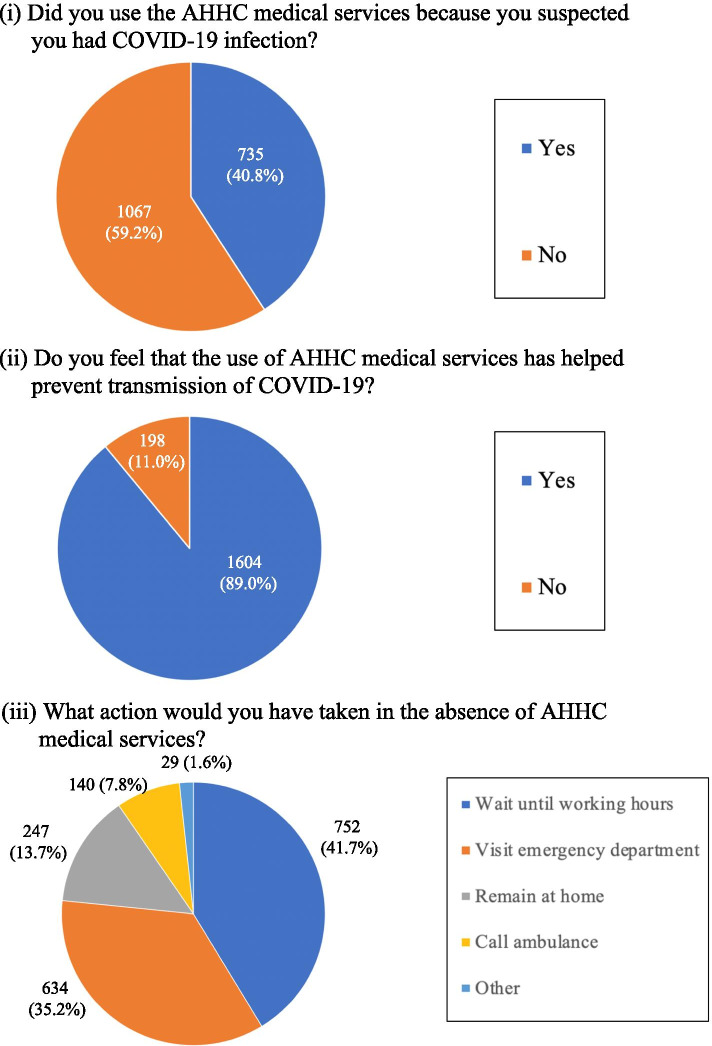
The results of the patient questionnaires after AHHC medical service use

Figure 1 displays the results of patient questionnaires after AHHC medical services. Of the responders, 40.8% used AHHC medical services because of suspected COVID-19. Furthermore, regardless of suspicion of COVID-19, the majority (89.0%) of responders who received AHHC medical services felt that AHHC medical services helped prevent transmission of COVID-19. A total of 774 out of 1802 (43.0%) considered that they would have visited an ED (634 patients, 35.2%) or called an ambulance (140 patients, 7.8%) if AHHC services were not used. On the other hand, 55.4% of responders answered that they would “remain at home” or “wait until working hour” if AHHC medical services were not available.

### Patient characteristics according to suspicion of COVID-19

Table 2 shows the patient characteristics based on whether the patients suspected themselves of having COVID-19 infection or not (based on the question “Did you use the AHHC medical services because you suspected you had COVID-19 infection?”). The patients with the following characteristics and the medical services to patients presenting symptoms were more likely to feel that their symptoms may be related to COVID-19: (i) age 16–74 years; (ii) orange triage color; (iii) mental/behavioral disorder or respiratory illness; (iv) mild symptom severity (a condition that could be treated with over-the-counter medications); (v) examination by health care personnel wearing PPE or screening with a diagnostic tool for COVID-19; and (vi) medical examination time of more than 26 min.

**Table 2:** Patient characteristics based on whether the patients suspected themselves of having COVID-19 infection or not^a^

	Whether the patients suspected themselves of having COVID-19 infection or not				
	Yes		No		p
	(n = 735) (40.8%)		(n = 1067) (59.2%)		
Age (years), n (%)					< 0.001
0–15	165	(26.7)	453	(73.3)	
16–64	495	(51.0)	475	(49.0)	
65–74	35	(40.7)	51	(59.3)	
> 74	40	(31.3)	88	(68.8)	
Telephone triage color, n (%)					< 0.001
Yellow	254	(35.2)	468	(64.8)	
Orange	425	(45.7)	505	(54.3)	
Red	56	(37.3)	94	(62.7)	
Diagnosis category, n (%)					< 0.001
Infectious and parasitic diseases	100	(28.9)	246	(71.1)	
Endocrine, nutritional, and metabolic diseases	4	(26.7)	11	(73.3)	
Mental and behavioral disorders	4	(50.0)	4	(50.0)	
Diseases of the nervous system	4	(12.9)	27	(87.1)	
Diseases of the ear and mastoid process	4	(12.9)	27	(87.1)	
Diseases of the circulatory system	1	(5.9)	16	(94.1)	
Diseases of the respiratory system	565	(57.4)	420	(42.6)	
Diseases of the digestive system	11	(14.3)	66	(85.7)	
Diseases of the skin and subcutaneous tissue	4	(10.3)	35	(89.7)	
Diseases of the musculoskeletal system and connective tissue	4	(7.8)	47	(92.2)	
Diseases of the genitourinary system	13	(20.3)	51	(79.7)	
Symptoms, signs, and abnormal clinical and laboratory findings, not elsewhere classified	8	(12.9)	54	(87.1)	
Injury, poisoning, and other certain consequences of external causes	10	(14.5)	59	(85.5)	
Other	3	(42.9)	4	(57.1)	
Symptom severity, n (%)					< 0.001
Mild	443	(45.2)	537	(54.8)	
Moderate	285	(36.4)	499	(63.6)	
Severe	7	(18.4)	31	(81.6)	
Personal protective equipment used, n (%)	557	(57.7)	408	(42.3)	< 0.001
Diagnostic tools for COVID-19 used, n (%)	377	(83.6)	74	(16.4)	< 0.001
Time of medical examination (minutes), mean (SD)	31.8	(14.7)	28.5	(13.6)	< 0.001
Time of medical examination (minutes), n (%)					< 0.001
< 15	83	(30.1)	193	(69.9)	
16–25	233	(37.3)	392	(62.7)	
≥ 26	419	(46.5)	482	(53.5)	

^a^Statistically significant variables are shown

*Abbreviations*: *AHHC* after hour house call, *ED* emergency department, *SD* standard deviation, *COVID-19* coronavirus disease 2019

### Patient characteristics according to patients’ perception on prevention of transmission of COVID-19

Table 3 shows the patients’ perception on the role of AHHC medical services in helping prevent the transmission of COVID-19 (based on the question “Do you feel that the use of AHHC medical services has helped prevent transmission of COVID-19?”), stratified by patient characteristics.

**Table 3:** Patients’ perception of prevention of transmission of COVID-19 stratified by patient characteristics^a^

	Do you feel that the use of AHHC medical services has helped prevent transmission of COVID-19?				
	Yes		No		p
	(n = 1604) (89.0%)		(n = 198) (11.0%)		
Age (years), n (%)					< 0.001
0–15	577	(93.4)	41	(6.6)	
16–64	839	(86.5)	131	(13.5)	
65–74	71	(82.6)	15	(17.4)	
> 74	117	(91.4)	11	(8.6)	
Diagnosis category, n (%)					< 0.001
Infectious and parasitic diseases	304	(87.9)	42	(12.1)	
Endocrine, nutritional, and metabolic diseases	15	(100.0)	0	(0.0)	
Mental and behavioral disorders	6	(75.0)	2	(25.0)	
Diseases of the nervous system	25	(80.6)	6	(19.4)	
Diseases of the ear and mastoid process	28	(90.3)	3	(9.7)	
Diseases of the circulatory system	15	(88.2)	2	(11.8)	
Diseases of the respiratory system	912	(92.6)	73	(7.4)	
Diseases of the digestive system	63	(81.8)	14	(18.2)	
Diseases of the skin and subcutaneous tissue	31	(79.5)	8	(20.5)	
Diseases of the musculoskeletal system and connective tissue	35	(68.6)	16	(31.4)	
Diseases of the genitourinary system	56	(87.5)	8	(12.5)	
Symptoms, signs and abnormal clinical and laboratory findings, not elsewhere classified	49	(79.0)	13	(21.0)	
Injury, poisoning, and other certain consequences of external causes	59	(85.5)	10	(14.5)	
Other	6	(85.7)	1	(14.3)	
Symptom severity, n (%)					< 0.001
Mild	898	(91.6)	82	(8.4)	
Moderate	680	(86.7)	104	(13.3)	
Severe	26	(68.4)	12	(31.6)	
Personal protective equipment used	895	(92.7)	70	(7.3)	< 0.001
Diagnostic tools for COVID-19 used	439	(97.3)	12	(2.7)	< 0.001
Time of medical examination (minutes), mean (SD)	30.1	(14.3)	27.2	(12.3)	0.007
Time of medical examination (minutes), n (%)					0.028
< 15	234	(84.8)	42	(15.2)	
16–25	554	(88.6)	71	(11.4)	
≥ 26	816	(90.6)	85	(9.4)	

^a^Statistically significant variables are shown

*Abbreviations*: *AHHC* after hour house call; *COVID-19* coronavirus disease 2019, *ED* emergency department, *SD* standard deviation

Patients under 15 years or over 75 years of age; with endocrine, nutritional, and metabolic diseases; with respiratory diseases; with diseases of the ear and mastoid process; examined by health care personnel wearing PPE; screened with diagnostic tools for COVID-19; and with a medical examination time of 26 min or more were more likely to feel that AHHC medical services prevented transmission of COVID-19. On the other hand, the group with severe symptoms (a condition that requires ambulance if not using an AHHC) had a higher proportion of “No” on whether AHHC medical services helped prevent transmission of COVID-19.

### Possible actions if AHHC medical services were not available

Based on the question “What action would you have taken in the absence of AHHC medical services”, patients aged 16–64 and 65–74 years were more likely to report that they would wait until working hours if AHHC medical services were not available than the overall proportion.

When patients suspected themselves of having COVID-19 infection, these patients were more likely to report that they would visit the ED or call an ambulance if AHHC medical services were unavailable. Conversely, these patients were less likely to report waiting until working hours if AHHC medical services were unavailable.

Even among the responders who indicated that they would remain at home or wait until working hours if AHHC medical services were not available, some patients had moderate (398/1802) or severe (13/1802) symptoms based on the physician’s assessment (Table 4).

**Table 4:** Possible actions if AHHC medical services were not available stratified by patient characteristics

	Expected actions if no use of AHHC medical services										
	Remain at home		Wait until working hours		Visit the ED		Call an ambulance		Other		
Characteristic	(n = 247) (13.7%)		(n = 752) (41.7%)		(n = 634) (35.2%)		(n = 140) (7.8%)		(n = 29) (1.6%)		p
Age (years), n (%)											< 0.001
0–15	70	(11.3)	250	(40.5)	276	(44.7)	14	(2.3)	8	(1.3)	
16–64	149	(15.4)	418	(43.1)	306	(31.5)	81	(8.4)	16	(1.6)	
65–74	9	(10.5)	38	(44.2)	23	(26.7)	13	(15.1)	3	(3.5)	
> 74	19	(14.8)	46	(35.9)	29	(22.7)	32	(25.0)	2	(1.6)	
Whether the patients suspected themselves of having COVID-19 infection or not											< 0.001
Yes	145	(13.6)	394	(36.9)	414	(38.8)	100	(9.4)	14	(1.3)	
No	102	(13.9)	358	(48.7)	220	(29.9)	40	(5.4)	15	(2.0)	
Symptom severity, n (%)											< 0.001
Mild	146	(14.9)	442	(45.1)	328	(33.5)	48	(4.9)	16	(1.6)	
Moderate	99	(12.6)	299	(38.1)	298	(38.0)	75	(9.6)	13	(1.7)	
Severe	2	(5.3)	11	(28.9)	8	(21.1)	17	(44.7)	0	(0.0)	

*Abbreviations*: *AHHC* after hour house call, *ED* emergency department

## Discussion

The present study found that (i) about 40% of the questionnaire responders used AHHC medical services because they suspected themselves of having COVID-19 infection; (ii) the majority of questionnaire responders who received AHHC medical services felt that these services can prevent transmission of COVID-19; (iii) and 43.0% of questionnaire responders considered that they would have visited an ED or called an ambulance if AHHC services were not available. This situation was more apparent if they suspected themselves of having COVID-19 infection. Furthermore, among those who used AHHC services between April 2020 and January 2021, half of the patients were examined by health care personnel wearing PPE, and about 20% of the patients were screened using diagnostic tools for COVID-19.

First, to the best of our knowledge, this is the first study to describe the characteristics of AHHC medical service users during the COVID-19 pandemic. Several patient characteristics and the medical services to patients presenting symptoms were associated with the patients’ perception of suspicion of COVID-19 infection, such as diagnosis of respiratory diseases, examination by health care personnel wearing PPE, and screening with a diagnostic tool for COVID-19. The AHHC medical services used PPE in patients with fever, and the presence of fever or respiratory symptoms may have been a factor in patients’ perception of suspicion of COVID-19. Previous studies have shown that the number of ED visits was reduced during the COVID-19 pandemic, even in cases of respiratory diseases and infections [20–22]. Therefore, patients with COVID-19 may use AHHC medical services. Indeed, 4.7% of patients were diagnosed with COVID-19.

Second, this survey revealed that most patients who were considered to require medical consultation by telephone triage felt that AHHC medical services were useful for preventing transmission of COVID-19. This result may be attributed to the fact that AHHC medical services provide consultations at home, using PPE when necessary, thus allowing patients to avoid contact with other patients in the hospital. A previous report also suggested that domiciliary radiography could reduce the risk of infected patients referring to family doctors’ offices or emergency departments and reduce transportation costs to the hospital [23].

Third, our results are similar to those of a previous study of AHHC medical service users in Australia, in which 40% felt that they would prefer ED visits or ambulance calls if AHHC services were not available [24]. This previous study concluded that AHHC medical services appear to be associated with a reduction in ED visits [24]. Furthermore, during the COVID-19 pandemic, several previous studies have raised concerns about the increased risk of delays in receiving medical care due to patients refraining from seeking healthcare services [1–4].

Our results also revealed that some patients indicated that they would remain at home or wait until working hours if AHHC medical services were not available despite having a condition that required an ED visit or an ambulance. These patients accounted for 22.8% of all respondents in the questionnaire (411/1802). This result may reflect the existence of a situation in which patients refrain from seeking medical services in Japan. AHHC medical services in Japan provide house calls with a vehicle equipped with medical equipment and can also provide medical procedures, such as the RT-PCR test for SARS-CoV-2, rapid SARS-CoV-2 antigen test, rapid influenza diagnostic test, drip test, X-ray, and ultrasonography. Therefore, our results suggest that AHHC medical services may be one of the strategies that can address the problem of patients avoiding visits to healthcare facilities during the COVID-19 pandemic. In addition, those who suspected themselves of having COVID-19 infection were more likely to intend to visit the ED or call an ambulance if AHHC services were not available. AHHC services may also be helpful in tackling the COVID-19 pandemic, including the ability to undergo medical examinations while preventing secondary infections.

We acknowledge that there are several limitations to our study. First, this study was based on information from only one private AHHC medical service provider. Therefore, users of this AHHC medical service provider may have different characteristics from non-users, resulting in selection biases. However, this provider was the first AHHC medical service provider in Japan and had the largest AHHC medical service market share in Japan. Furthermore, before providing AHHC medical services, the provider conducted telephone triage to determine the necessity of medical services so as to provide medical services only to those considered to require medical consultation. Second, the response rate of the patient questionnaire in this study was relatively low (11.3%), which may have led to selection bias. For example, there were differences in the proportion of patients’ age, drug prescriptions, examination by health care personnel wearing PPE, and use of diagnostic tools for COVID-19 between responders and non-responders. Third, although about 40% felt that they would visit the ED or call for an ambulance if they could not use AHHC medical services, it is not clear how many patients refrained from seeking healthcare services. Further study is required to evaluate AHHC medical services in the context of overall after-hours care because our results were based on only the patient's impression who used AHHC medical services.

## Conclusion

The majority of patients who received AHHC medical services felt that these services can help prevent transmission of COVID-19 because of the medical services provided in their own homes. AHHC medical services may be one of the strategies for alleviating patients’ hesitation to visit healthcare facilities and may reduce the risk of delayed hospital visits during emergencies.

## Data Availability

The data that support the findings of this study are available from Fast Doctor, Ltd., but restrictions apply to the availability of these data, which were used under license for the current study, and so are not publicly available.

## Abbreviations

AHHC: After-hour house-call
COVID-19: Coronavirus disease 2019
ED: Emergency department
PPE: Personal protective equipment
SARS-CoV-2: Severe acute respiratory syndrome coronavirus 2
